# A Near-Chromosome Level Genome Assembly of *Anopheles stephensi*

**DOI:** 10.3389/fgene.2020.565626

**Published:** 2020-11-16

**Authors:** Afiya Razia Chida, Samathmika Ravi, Suvratha Jayaprasad, Kiran Paul, Jaysmita Saha, Chinjusha Suresh, Saurabh Whadgar, Naveen Kumar, Raksha Rao K, Chaitali Ghosh, Bibha Choudhary, Suresh Subramani, Subhashini Srinivasan

**Affiliations:** ^1^Institute of Bioinformatics and Applied Biotechnology, Bangalore, India; ^2^Tata Institute for Genetics and Society Center at inStem, Bangalore, India

**Keywords:** homology-based assembly, simulated mate-pair, comparative genomics, genome browser, olfactory receptors, cytochromeP450, gene expression profile, developmental transcriptome

## Abstract

Malaria remains a major healthcare risk to growing economies like India, and a chromosome-level reference genome of *Anopheles stephensi* is critical for successful vector management and understanding of vector evolution using comparative genomics. We report chromosome-level assemblies of an Indian strain, STE2, and a Pakistani strain SDA-500 by combining draft genomes of the two strains using a homology-based iterative approach. The resulting assembly IndV3/PakV3 with L50 of 9/12 and N50 6.3/6.9 Mb had scaffolds long enough for building 90% of the euchromatic regions of the three chromosomes, IndV3s/PakV3s, using low-resolution physical markers and enabled the generation of the next version of genome assemblies, IndV4/PakV4, using HiC data. We have validated these assemblies using contact maps against publicly available HiC raw data from two strains including STE2 and another lab strain of *An. stephensi* from UCI and compare the quality of the assemblies with other assemblies made available as preprints since the submission of the manuscript. We show that the IndV3s and IndV4 assemblies are sensitive in identifying a homozygous 2Rb inversion in the UCI strain and a 2Rb polymorphism in the STE2 strain. Multiple tandem copies of CYP6a14, 4c1, and 4c21 genes, implicated in insecticide resistance, lie within this inversion locus. Comparison of assembled genomes suggests a variation of 1 in 81 positions between the UCI and STE2 lab strains, 1 in 82 between SDA-500 and UCI strain, and 1 in 113 between SDA-500 and STE2 strains of *An. stephensi*, which are closer than 1 in 68 variations among individuals from two other lab strains sequenced and reported here. Based on the developmental transcriptome and orthology of all the 54 olfactory receptors (ORs) to those of other *Anopheles* species, we identify an OR with the potential for host recognition in the genus *Anopheles*. A comparative analysis of *An. stephensi* genomes with the completed genomes of a few other *Anopheles* species suggests limited inter-chromosomal gene flow and loss of synteny within chromosomal arms even among the closely related species.

## Introduction

There are ∼450 recorded species in the genus *Anopheles* with roughly 100 being vectors of malaria in various endemic regions in India, Africa, and elsewhere. Efforts to obtain reference genome sequences for various vectors that spread malaria by transmitting *Plasmodium* are under way. Chromosome level assemblies of *Anopheles gambiae* and *Anopheles funestus*, both malaria vectors from Africa, have been reported (Sharakhova et al., 2007; Ghurye et al., 2019a). Draft genomes of an *Anopheles stephensi* strain from India (Jiang et al., 2014) and sixteen other *Anopheles* genera (Neafsey et al., 2015) have also been reported.

*Anopheles stephensi* is an urban species of mosquito that is responsible for causing roughly 12% malaria in India. The major genome resource so far available for *An. stephensi* is the draft genome reported in 2014 (Jiang et al., 2014). In that report, a number of sequencing technologies including Pacbio, 454, Illumina, and BAC-end sequencing were used to generate the draft assembly. The 454 reads were sequenced from multiple mate-pair libraries and include 12.2X coverage from single-end reads, 2.2X coverage from 3-kilobase (kb) paired-end reads, 3.4X coverage from 8-kb paired-end reads, and 1.7X coverage from 20-kb paired-end reads. The majority of 454 reads were in the range of 194–395 base-pairs (bp) in length. Illumina short reads with a coverage of 86.4X, read length of 101 bp paired-end reads, and an average insert size of approximately 200 bp were sequenced. Also, ten cells of PacBio reads using RS1 sequencing for male genomic DNA produced 5.2X coverage with a median read length of 1295 bp. A hybrid assembly, combining 454 and Illumina data, was further improved by filling the gaps with error-corrected PacBio reads and scaffolding using BAC-ends with an insert size of ∼140 kb. The resulting assembly contained 23,371 scaffolds spanning 221 Mb, including 11.8 Mb (5.3%) of gaps filled with Ns (unspecified nucleotides). The L50 of the assembly was 37. The N50 scaffold size was 1.59 Mb, and the longest scaffold was 5.9 Mb. Considering that the assembly included various sequencing technologies and strategies, the assembly has the least bias from the use of particular technology platforms.

More recently, draft genome assemblies of 16 diverse species of *Anopheles* mosquitoes was generated, including a strain of *An. stephensi* from Pakistan (SDA-500) (Neafsey et al., 2015). The contigs for the Pakistani strain from this report were produced using short-read sequences using an Illumina sequencer, with libraries ranging from small to medium and large insert sizes having a coverage of about 100X and read length of 101 bp. From this, the scaffold level assembly for the Pakistani strain of *An. stephensi* was reported by assembling reads with ALLPATHS-LG (P) (Neafsey et al., 2015). The study reported an N50 of 0.8 Mb and the L50 of 85.

Yet another valuable resource for *An. stephensi* is the low-resolution physical map of the chromosomes (Sharakhova et al., 2010). This study reported a physical map consisting of 422 DNA markers hybridized to 379 chromosomal sites of the *An. stephensi* polytene chromosomes providing a resolution of 0.6 Mb. Of these, 241 are cDNA markers for which both locations and sequences are accessible.

In the past, experimentally derived mate-pair libraries of varying insert sizes have been used in scaffolding useful draft genomes for many species using contigs built from short paired-end reads. Tools like SOAPdenovo use reads from mate-pair libraries to connect contigs from short paired-end reads into scaffolds based on the insert size information to create hundreds of draft genome assemblies whose quality was mainly proportional to the insert size of the mate-pair libraries. It should be mentioned here that the gap between contigs measured by insert size of a given mate-pair read anchoring the two contigs is filled with Ns. However, the advent of cost-effective long-read sequencing technologies has made the creation of mate-pair libraries, an arduous step, obsolete. Long reads, along with technologies such as HiC, are generating high-quality reference genomes of many non-model organisms. This has created an opportunity to provide chromosomal context to draft scaffolds, the holy grail of all genome assemblies for other strains, cultivars, and landraces at reduced cost.

Reference-guided improvement of draft genome assembly of individuals from the same species is becoming routine. Mate-pair libraries from one *Arabidopsis thaliana* strain were shared across many strains to build super-scaffolds for all individuals (Schneeberger et al., 2011). Also, assisted assembly of closely related species significantly improved the contiguity of low-coverage mammalian assemblies (Gnerre et al., 2009). For example, the draft genomes of four species including bush baby, African elephant, rabbit, and guinea pig from the “Mammal24 – 2X” project were built using both human and canine references (Gnerre et al., 2009). Reference-based assembly relies on DNA-level homology between the reference and the draft genomes, which can only be expected if both are from the same species. In the absence of a reference genome from the same species, a draft assembly can be improved using the synteny and protein-level homology between species to provide chromosomal context to scaffolds. Recently, a chromosome-level genome of *Lates calcarifer* was assembled from a draft genome using long-read sequencing, transcriptome data, optical/genetic mapping, and synteny to two closely related seabasses (Vij et al., 2016). In yet another report, 16 out of 60 chromosomes of the Tibetan antelope were reconstructed from draft assemblies using its homology to cattle (Kim et al., 2013). In fact, using independent mapping data and conserved synteny between the cattle and human genomes, 91% of the cattle genome was placed onto 30 chromosomes (Zimin et al., 2009). In a review article, synteny has been used to filter, organize, and process local similarities between genome sequences of related organisms to build a coherent global chromosomal context (Batzoglou, 2005). Similarly, the malarial strain, *Plasmodium falciparum* HB3, was improved using the reference of *P. falciparum* 3D7 combined with an assisted assembly approach that significantly improved the contiguity of the former (Gnerre et al., 2009). More recently, it has been shown that the scaffolds from draft genomes of 20 *Anopheles* species could be improved considerably by providing chromosomal context using synteny at the gene level (Waterhouse et al., 2020).

Here, we have created chromosome level assemblies of *An. stephensi* for two strains including STE2 lab strain from Delhi and a Pakistan strain SDA-500 by combining draft assemblies of these two strains using both linearity of physical markers (Jiang et al., 2014) and HiC data for two strains.

## Results

### Homology-Based Assembly and Pseudomolecule Generation

**FIGURE 1:**
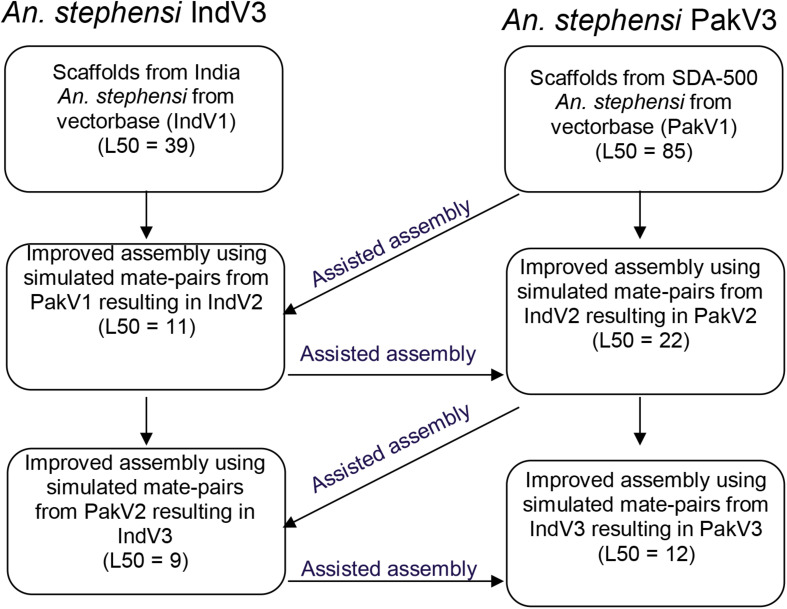
Schematic diagram showing the iterative approach followed to improve the assemblies of both strains of *An. stephensi*.

We utilized contigs/scaffolds from draft assemblies of two different strains of *An. stephensi* from public repositories to iteratively improve assemblies of both using complementary information from one to the other as shown in Figure 1. Table 1 describes the assembly metrics through the iterative improvement process. The initial assembly of Indian strain STE2 (called IndV1 here) downloaded from VectorBase had a L50 of 37, and that for the Pakistani strain SDA-500 (called PakV1 here) was 85. In the first iteration, the L50 of IndV1 was reduced to 11 (IndV2) with the help of simulated mate pairs of varying insert sizes from PakV1. During the second iteration, the L50 of PakV1 was reduced to 22 (PakV2) using the simulated mate pairs of varying insert sizes from IndV2. The subsequent iteration resulted in assemblies with the L50 of the STE2 strain dropping to 9 from 37 and that for the SDA-500 strain to 12 from 85. Further iteration did not show significant improvement, suggesting saturation of complementing information in the initial draft assemblies of the two strains.

**TABLE 1:** Homology-based iterative assembly.

**Iterations/strain**	**IndV1/PakV1**	**IndV2/PakV2**	**IndV3/PakV3**	**IndV4qm using HiC/PakV4**	**STE2/SDA-500 (Waterhouse et al., 2020)**	**IndV3s/PakV3s stitched**
Longest scaffold (Mb)	5.9/3.4	21.3/9.8	24.3/17.1	47.5/48.9	10.2/7	58/62.5
L50	37/85	11/22	9/12	3/4	16/38	2/2
N50* (Mb)	1.7/0.8	5.09/3.18	6.5/6.9	39.3/18.9	4.19/1.7	43/51.4
Number of scaffolds	7806/1110	7661/770	7657/742	7423/638	7677/873	5/5
Assembly size (Mb)	210/225	213/230	213/244	220/244.4	210/225	180/193.5
Percent Ns	5.62/12.95	6.9/14.88	6.91/19.71	7.14/19.73	5.6/12.9	6.44/16.93

Assembly metrics for Indian (STE2) and Pakistani (SDA-500) strains after every iteration. *N50 is the scaffold length, x, such that 50% of the genome is assembled on scaffolds of length x or longer.

**FIGURE 2:**
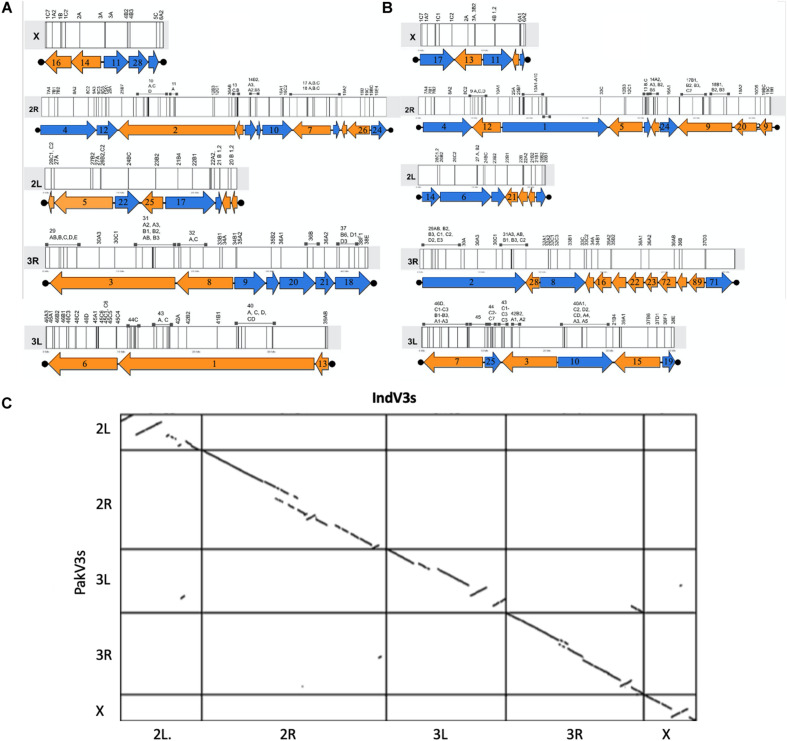
Generation of pseudomolecules. **(A,B)** Karyogram showing placement of scaffolds from IndV3 (top-left) and PakV3 (top-right) assemblies used to stitch chromosome level assemblies, IndV3s and PakV3s, with DNA marker location on scaffolds. **(C)** Dot plot of IndV3s against PakV3s.

In the absence of HiC and/or optical mapping data, the published physical mapping data was used to order the scaffolds and stitch them into pseudomolecules to enable comparative genomics. It is important to note here that the scaffolds were long enough for super scaffolding using the placement of 230 out of the 241 cDNA markers (Sharakhova et al., 2010) for which the sequences were available (Jiang et al., 2014). Out of 230 markers, 209 (90%) could be mapped with confidence to scaffolds. Supplementary Figure S1 shows the overlap between sequences from the physical mapping information for *An. stephensi* and the scaffolds generated from the third iteration of homology-based assembly for both the STE2 (IndV3) and SDA-500 (PakV3) strains of *An. stephensi*. The almost null overlap of physical maps placed on scaffolds helped in uniquely assigning chromosomes to scaffolds, as shown in Supplementary Figure S1a. However, physical markers from multiple arms hit a few scaffolds challenging the assignment of chromosomes for some long scaffolds (see Supplementary Table S1), which were subsequently resolved as described in section “Materials and Methods.” In some other cases, very short scaffolds, which had only one physical marker, could not be assigned the right orientation appearing like pseudo inversions in the dot plot (Figure 2C). The karyogram in Figure 2 shows the final order and orientation of the physical markers on each chromosome after building the pseudomolecules for IndV3s/PakV3s scaffolds. The total bases incorporated into pseudomolecules after stitching constituted 180 Mb for IndV3s and 193 Mb for PakV3s.

The assembly statistics at various steps during homology-based scaffolding, along with the final IndV3/PakV3 assemblies, are compared in Table 1, which includes scaffolds from Waterhouse et al. (2020) for these two strains and IndV4/PakV4 assemblies reported here using the HiC data on IndV3/PakV3 scaffolds by SALSA (Ghurye et al., 2019b) as described in the next section. The L50 and N50 values for IndV3/PakV3 are significantly better than those reported for STE2/SDA-500 strains using synteny (Waterhouse et al., 2020).

### Completeness and Annotation

We evaluated the overall completeness of the two assemblies, IndV3s and PakV3s, using diverse approaches. These include (i) percentage of physical markers aligning with the scaffolds, (ii) total predicted proteins, (iii) a set of elite proteins from BUSCO (Supplementary Table S2), and (iv) synteny to other *Anopheles* genomes (Supplementary Figure S7). Additionally, we assessed the gene structures for a set of predicted genes from IndV3s to validate the quality of assembly with respect to gene loci (Supplementary Table S3).

The number of physical markers mapped to each assembly, 87% to IndV3s and 84% to PakV3s, provides a macroscopic measure of the completeness of each chromosome. For example, 15/18 markers (83%) from the X chromosome mapped to the IndV3 assembly and 84/88 (95%) from 2R, 37/40 (92%) from 3R, 52/56 (92%) from 3L, and 21/28 (75%) markers from 2L were mapped to IndV3 assembly. Furthermore, a total of 180 and 193 Mb of scaffolds from IndV3 and PakV3 (Supplementary Figure S1b) scaffolds could be placed on the chromosomes using physical markers to stitch the IndV3s and PakV3s assemblies.

Gene prediction using Augustus with *Aedes aegypti* as a model resulted in the prediction of 21,378 and 20,083 proteins for IndV3s and PakV3s, respectively. These are comparable to the 20,318 proteins predicted in-house from the high-resolution, chromosome-level assembly of *An. funestus* using exactly the same method. The proteins were validated using both publicly available transcriptome data from *An. stephensi* and orthology to the proteomes of other species. For transcriptome-based validation, midgut transcriptome and the Indian peptide database from VectorBase were used, and for orthology, the predicted genes from other available *Anopheles* genera in Swiss-Prot and TrEMBL were used. Supplementary Figure S2 depicts the intersection of predicted proteins for IndV3s (S2a) and PakV3s (S2b) validated by each database. Of the 21,378 genes predicted for IndV3s, 12,148 were validated using one and/or more of the databases. For PakV3s, 11,303 of the 20,083 proteins predicted by Augustus were validated using these databases. An annotated GTF file is made available via the genome browser site listed under the section “Data Availability Statement.”

The completeness of the proteome in IndV3s and PakV3s assemblies was also assessed with BUSCO, which utilizes evolutionarily informed expectations of gene content. Out of the total of 1013 complete single-copy elite marker genes for *Arthropoda* used by BUSCO, 90.2% were placed on chromosomes and 98.2% in all scaffolds of IndV3s, which is a very good indication of the completeness of the assembly.

To assess the completeness of assembly across gene loci, we assessed gene structures of representative, predicted genes of interest to vector biologists across IndV3s. These include genes that are implicated in insecticide resistance, parasitic infection, and other DNA break-repair mechanisms, as listed in Supplementary Table S3. Based on the well-studied orthologs, we found that the gene structure for all the genes of interest (Supplementary Table S3) is predicted at full length. The links listed for Augustus ID in Supplementary Table S3 can be used to view the gene structure on the browser and download sequences. For example, all 20 exons are predicted intact for the *KDR* gene with exon structures and sizes similar to orthologs from other species (Supplementary Figure S3). With the exception of the *MRE11* gene, all are found in assembled chromosomes. The protein sequences of the genes are given in Supplementary Text S1. The accessions of the query protein sequences used for the identification of orthologs in *An. stephensi* are given in Supplementary Table S4.

### Validation of Homology-Based Scaffolding

**FIGURE 3:**
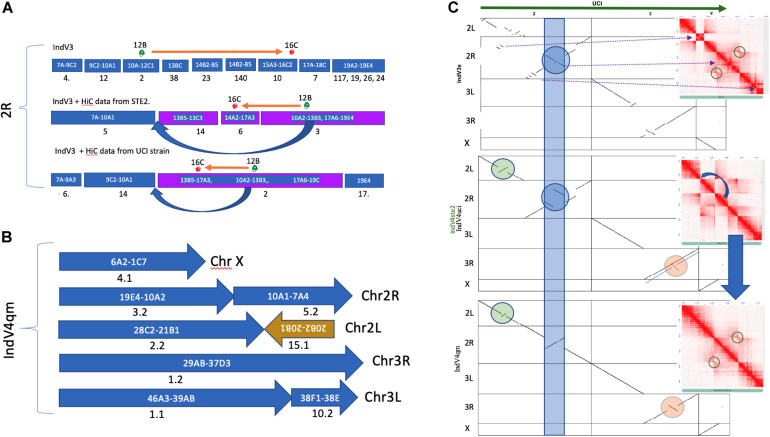
Homology-based scaffolding. **(A)** Scaffolds after homology-based scaffolding covering the range of physical markers for only 2R. **(B)** Karyogram of IndV4. **(C)** Dot plot of UCI assembly against all assemblies of the STE2 strain reported here including IndV3s (top), IndV4uci/IndV4ste2 (middle, only 3R is different), and IndV4qm (bottom). Inset shows contact maps of the 2R arm with HiC reads from the STE2 strain against the respective STE2 assemblies. The blue curved arrow shows the manually moved block. The blue strip, green, and peach circles highlight potential inversions against UCI assembly in respective STE2 assemblies.

The quality and statistics of the IndV3 assembly depend on two factors including homology-based scaffolding and resolution of the physical markers. In order to validate the homology-based scaffolding method, we scaffolded IndV3 directly using HiC data available in the public domain (Chakraborty et al., 2020; Lukyanchikova et al., 2020) using SALSA. The two assemblies generated using HiC data from the UCI (called IndV4uci) and STE2 (called IndV4ste2) strains, respectively, on IndV3 scaffolds, resulted in scaffolds with very similar linearity of physical markers as shown in Figure 3A. Merging these two assemblies using QuickMerge (Chakraborty et al., 2016), we obtained an assembly, IndV4qm, with a N50 value of 39 Mb and L50 of 3. The orientations of many short scaffolds from IndV3 with unassigned orientation in IndV3s (Figure 3C) are assigned the correct orientations in IndV4qm (Figure 3B). However, near the potential 2Rb inversion loci (blue band in Figure 3C), IndV4uci/IndV4ste2 both suggest a translocation that appears to mimic the inversion of the two 2Rb breakpoints, near the markers 12B and 16B (marked above the mock karyogram in Figure 3A). Figure 3C-middle shows both the dot plot of IndV4uci/ste2 assemblies against the UCI assembly and the contact map of the 2R arm of the two IndV4uci/ste2 generated with HiC data from STE2, which clearly rules out a translocation but provides accurate coordinates for the breakpoints, which could easily be corrected, as shown in Figure 3C-bottom. The inversion breakpoints are circled around the butterfly in the respective contact maps. The block that was moved manually is shown with a blue arrow in Figure 3C-middle and under the karyogram in Figure 3A. Figure 3C-bottom shows the dot plot of corrected IndV4qm assembly against UCI assembly with a contact map against HiC data from the STE2 strain. In IndV4qm, we observe an inversion in the center of the 2L arm around marker 24 (circled green in Figure 3C) and the 3R arm around markers 31–33 (circled peach in Figure 3C) with respect to UCI assembly. Interestingly, the 3R inversion is not seen in IndV4uci but only in IndV4ste2 as shown by the overlapping lines in the dotplot in Figure 3C-middle. To our knowledge, there is no report of breakpoint pairs at these positions. These inversions are part of very long scaffolds assembled using HiC contact and could potentially represent novel rare inversions. Supplementary Figure S9 provides the coordinates for these breakpoints in IndV4qm assembly using the contact map against HiC from the STE2 strain.

At the scaffold level, one of the scaffolds from IndV3 clearly shows the advantage of the method proposed here compared to that of Waterhouse et al. (2020). Scaffold18 in IndV3 and scaffold19 in PakV3 with lengths of 3.9 and 4.2 Mb, respectively, span the centromere of chromosome 3. This scaffold includes markers from both 3R and 3L arms, including 61_G06_BU039005_3R_37_B(6), 19_D07_BU038946_3R_37_ D(1), 211A05_EX227515_3R_37_D(1), 627112_BM636978_ 3R_37_D(3), 211B11_EX227529_3L_38_F(1), and AsHyp16_ AY162228_3L_38_E.

A perfect synteny of scaffold18 from IndV3 was seen across the centromeric region of the high-quality assembly of chromosome 3 from the UCI strain (see Supplementary Figure S4). This scaffold was not present in the input scaffolds from IndV1. In IndV1, this region is split into three scaffolds including scaffold_00065, scaffold_00059, and scaffold_00062. Also, even in the scaffolds reported for the STE2 strain by Waterhouse et al. (2020), the centromere region is split into 3R and 3L arms with two scaffolds including ASTEI_SS000034 and ASTEI_SS000035.

### Validation of Assemblies

**FIGURE 4:**
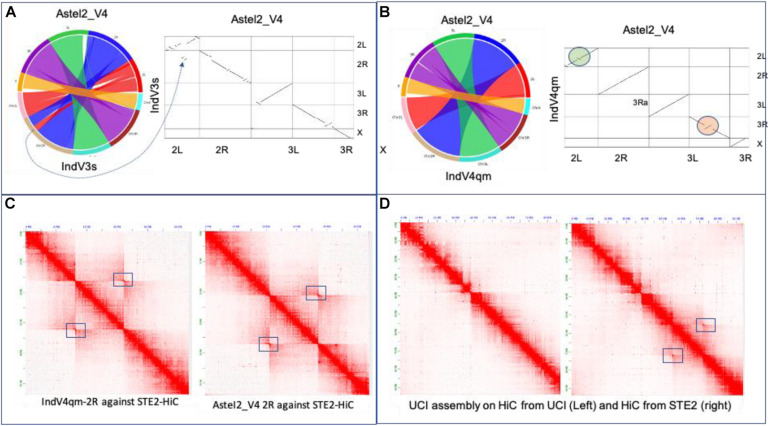
Validation of assemblies. **(A)** Synteny and dot plot of IndV3s against AsteI2_V4. **(B)** Synteny and dot plot of IndV4qm against AsteI2_V4 assembly. **(C)** Contact maps for 2R of IndV4qma (left) and AsteI2_V4 (right) using HiC data from the STE2 strain. **(D)** Contact maps of chromosome 2 of the UCI assembly against the two HiC datasets for the UCI and STE2 strains.

We have compared various chromosome-level assemblies reported here to the two other assemblies reported via preprint since the submission of this manuscript. The dot plot in Figure 4A showing synteny between IndV3s and AsteI2_V4 assemblies (Lukyanchikova et al., 2020) shows a high level of synteny, except for a few inverted regions in IndV3s stemming from placement of scaffolds with unassignable orientation appearing as pseudoinversions. The dot plot in Figure 4B between IndV4qm and AsteI2_V4 assemblies shows perfect match in chromosomal arms 3L and X, without the need for any manual correction. The only manual correction performed was within the inversion locus in 2R stemming from conflicting contacts within the HiC data from 2Rb heterozygosity. The UCI assembly clearly has 2Rb inversion within the reported assembly, as seen in the dot plot against IndV3s (Figure 3C-top) and IndV4qm (Figure 3C-bottom), which are consistent with the linearity of the physical markers.

Figure 4C shows the contact map for the 2R arm generated using IndV4qm (left) and AsteI2_V4 (right) using HiC from the STE2 strain, suggesting presence of the 2Rb inversion heterozygosity within the HiC contacts. Figure 4D shows contact maps of chromosome 2 of the UCI genome assembly against HiC data generated from the UCI (left) and STE2 strains (right), respectively. Since the UCI assembly has the 2Rb inversion and shows no off-diagonal butterfly pattern in Figure 4D-left, it can be concluded that the UCI strain is homozygous for the 2Rb inversion. In contrast, the off-diagonal butterfly pattern in the contact map in Figure 4D-right produced using UCI assembly with HiC from the STE2 strain is suggestive of the presence of a haplotype in the STE2 strain that is collinear with respect to the physical markers. Since HiC data from the STE2 strain displays butterfly patterns against genome assemblies with and without the 2Rb inversion, such as UCI (Figure 4D-right) and IndV4qm (Figure 4C-left) assemblies, it can be concluded that the STE2 strain is polymorphic for the 2Rb inversion as suggested in the preprint (Lukyanchikova et al., 2020).

### Genes in the 2Rb Inversion Locus

**FIGURE 5:**
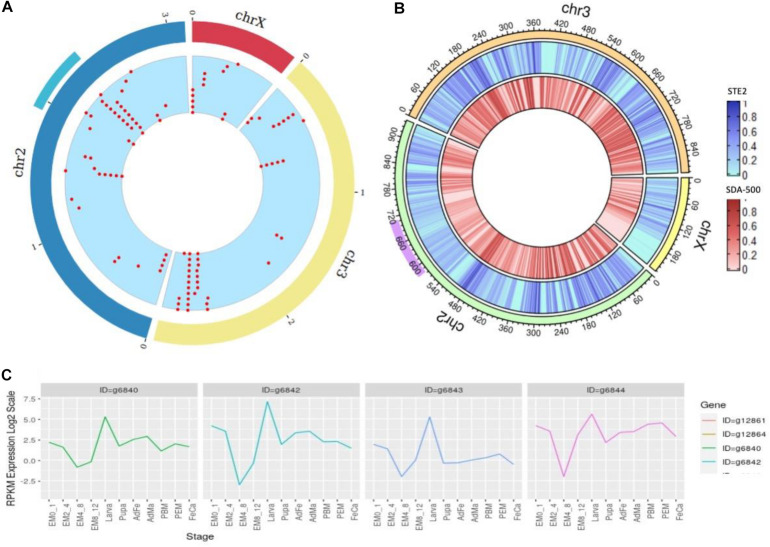
**(A)** Density of cytochrome genes across the genome of *An. stephensi* UCI assembly, the cyan arc representing the 2Rb inversion locus and the red dots representing locations of cytochrome P450 genes across the genome. **(B)** SNP density at a window of 10,000 bases within STE2 (blue) and SDA-500 (red) strains compared to UCI strain (right) with purple arc showing 2Rb inversion locus. **(C)** Developmental expression profiles of the four copies of the CYP6a14 gene.

There are 1000+ genes within this locus ranging from 58.4 to 70.7 Mb in the UCI assembly. As the 2Rb inversion polymorphism in *An. stephensi* has been associated with resistance (Chakraborty et al., 2016) to insecticides, we looked for cytochrome P450 genes within this locus that confer metabolic resistance. Figure 5A shows two major cytochrome P450 clusters in the *An. stephensi* genome, one being within the 2Rb inversion locus (Figure 5A cyan arc) and the other at the tail end of chromosome 3.

We used genome assembly from the UCI strain to study cytochrome P450 genes in this locus (Table 2), as this strain is homozygous for this inversion. The 2Rb locus has multiple copies of a few cytochrome P450 genes including CYP6a14 (four copies), CYP4c1 (five copies), CYP4d2, CYP6a2, and CYP4c21 (three copies), with many reported to be implicated in insecticide resistance (Amichot et al., 2004; Lien et al., 2019). In Table 2, the 2nd and 3rd columns are gene IDs of respective cytochrome P450 genes predicted by Augustus on the UCI assembly and IndV3s assembly. Interestingly, homologs of all these genes are found in IndV3s.

**TABLE 2:** List of cytochromes P450 genes within the 2Rb inversion locus in both the UCI and the IndV3 assemblies.

**CypP450 genes**	**UCI ID**	**IndV3s ID**
4c1	3760, 4551, 4615, 4614, 4624	5865, 6996, 7096, 7095, 7108
4d2	3996, 3994, 3998	6192, 6192, 6183
4d14	3995	6192
6a13	4446	6841
6a2	4444, 4451, 4449	6839, 6845, 6843
6a14	4445, 4448, 4450, 4447	6840, 6843, 6844, 6842
CYP-1	4512	6919
313a4	4616	7097
4c21	4626, 4622, 4625	7110, 7105, 7109

The expression profile of the four copies of CYP6a14 from the 2Rb inversion locus was analyzed using the transcriptome data available for many developmental stages shown in Supplementary Table S7. All the four genes are upregulated in larva stage (Figure 5C), as expected for genes that offer resistance to insecticide. The four copies of CYP6a14 genes are divergent at the DNA level, as seen by the variation in the expression profile, but have significant homology at the protein level.

### Genetic Diversity Among Various Lab Strains

We compared the assembled genomes of strains STE2 (IndV4) and SDA-500 (PakV4) with that from the UCI strain, to assess the level of diversity between the two lab strains by aligning the stitched IndV4/PakV4 assemblies against the UCI assembly using minimap2 (Li, 2018). We find 1 in 90 positions varying between the STE2 and UCI strains, 1 in 82 between PakV4s (SDA-500) and the UCI strain compared to 1 in 113 between IndV4s (STE2) and PakV4s (SDA-500). Figure 5B shows the SNP density for STE2 and SDA-500 strains on UCI strains. The 2RB inversion loci shown in purple in Figure 5B has relatively fewer SNPs compared to the rest of the genome. The 3L and 2L arms are relatively high SNP density among the three strains.

In order to check if the diversity among the 3 strains, UCI, STE2, and SDA-500, is consistent with the geographical separation of individuals, we have also sequenced 30X coverage of six lab female mosquitoes originating from one urban city and one male individual from another city. Supplementary Table S5 gives the SNP density, and S6 shows the mapping percentage of the sequenced individuals. The number of variants in these individuals with respect to the IndV3 reference is roughly 2.6 million, suggesting an overall SNP density of 1 in every 68 bases of the genome. This is ten times higher than the number of SNPs (319,751) reported using diverse individuals from a lab strain (Jiang et al., 2014), which may be because the individuals were taken from the same colony that was used in creating the reference. The X chromosome has the lowest SNP density with 1 in 85, which is in tune with what is reported for *An. gambiae* and *An. stephensi* (Holt et al., 2002; Jiang et al., 2014). In Supplementary Table S5, we show SNP density for the X chromosome from both female and male individuals to make sure the lower SNP density in X is not resulting from lower coverage of X from the hemizygous male. In females, the SNP density is at 1 in 82 bases and is only slightly higher than the SNP density of 1 in 114 bases in males.

### Evolution of Olfactory Receptors

**FIGURE 6:**
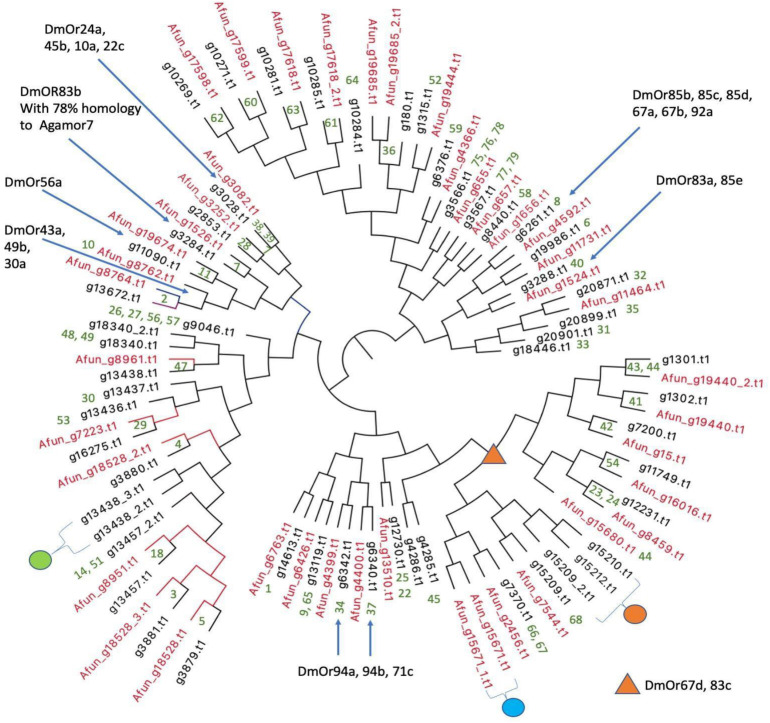
Phylogenetic relationships of olfactory receptors. The 54 ORs of *An. stephensi* (black) predicted from IndV3s with the 42 ORs predicted for *An. funestus* (red). Embedded numbers in green are the accession IDs of *An. gambiae* ORs. The green dot represents ORs missing in *An. funestus* and expanded into five ORs in *An. gambiae* including accessions 13, 15, 16, 17, 55. The cyan dot represents ORs missing in *An. stephensi*, duplicated in tandem in *An. funestus* and duplicated in *An. gambiae* with accessions 43–44. The orange dot is missing in *An. funestus* and expanded into six ORs in *An. gambiae* with accession IDs of 69–74. The blue arrows and orange triangle show ORs of *D. melanogaster* based on their orthology to *An. gambiae* reported elsewhere (Hill et al., 2002).

Olfactory receptor genes (ORs) are of interest from an evolutionary point of view in vectors because of their role in host choice and therefore disease transmission. The numbers of ORs in different vectors vary widely. *Ae. aegypti* has 110 ORs (Bohbot et al., 2007) whereas *An. gambiae* encodes for only 79 ORs (Hill et al., 2002). The total number of OR proteins predicted from IndV3s that are orthologous to *An. gambiae* ORs is 54, which matches with the number of ORs predicted from the high-quality UCI assembly (not shown). The protein sequences of the 54 ORs from *An. stephensi* and 42 from predicted *An. funestus* (Ghurye et al., 2019a; Supplementary Text S2) are used to create a phylogenetic tree (Figure 6). The corresponding orthologs of *An. gambiae* ORs are in green at the branch points. Supplementary Figure S5 shows a phylogenetic tree including orthologs of ORs from all three *Anopheles* species.

In all three species, some orthologous ORs appear in tandem in the genome, as reported previously (Jiang et al., 2014). For example, the three ORs from *An. stephensi* with accession IDs of (i) g13438 with orthology to OR 47 of *An. gambiae* and 8961 of *An. funestus* and (ii) g13438_2, and (iii) g13438_3 in tandem are missing in *An. funestus* (see green dot in Figure 6) but expanded into six ORs in *An. gambiae* with accessions of 13, 15, 16, 17, and 55. The orthologs of ORs 46 and 47 from *An. gambiae* are missing in *An. stephensi* but are present in tandem in *An. funestus* (cyan dot in Figure 6). The orthologs of *An. stephensi* with accessions of g15209_2, g15210, and g15212 (orange dot in Figure 6) are missing in *An. funestus* but are collinear orthologs of six ORs in *An. gambiae* with accessions ranging from 69 to 74 (see highlighted in orange in Supplementary Figure S5). There are orthologs for five *An. gambiae* ORs with accession IDs of 12, 19, 20, 21, and 50 missing in both *An. stephensi* and *An. funestus*, which cluster into a group (see highlighted in green in Supplementary Figure S5) and is also reported missing in *Drosophila melanogaster* (Holt et al., 2002).

A majority of the 54 OR genes in *An. stephensi* show a higher expression in early embryonic stages (Supplementary Figure S6). Interestingly, the relatively lower number of ORs that are upregulated in other stages is not upregulated in embryonic stages. The three ORs, g10271, g10269, and g3284, of *An. stephensi*, which are highly expressed in pupa are orthologous to genes GRPor60, GPRor62, and GPRor7 from *An. gambiae*. The gene g13672 of *An. stephensi with* increasing expression from pupa to adult female to adult male is an ortholog of GORor2 from *An. gambiae* and DmOR43a of *D. melanogaster* that has a known ligand with potential function in host recognition by binding to cyclohexanone (Carlson, 2001).

## Discussion

Here, we report chromosome-level assemblies of multiple strains (*STE2 and SDA-500*) of *An. stephensi*, a major malaria-causing vector in urban India. Draft assemblies of two strains of *An. stephensi* were obtained from VectorBase, and their complementarity was used to improve the assembly of each strain using simulated mate pairs from the other in an iterative fashion. The L50 for the STE2 strain improved from 37 to 9, and that for the SDA-500 strain improved from 85 to 12 after three iterations. The longest scaffolds increased in lengths from 6 to 24 Mb and N50 from 1.9 to 6.3 Mb for the STE2 strain. Table 1 compares the statistics with those reported elsewhere.

In order to validate the method proposed here, the scaffolds from the IndV3 assembly were further scaffolded using the recently available HiC data from two strains (Chakraborty et al., 2020; Waterhouse et al., 2020) to generate an assembly with a L50 of 3, N50 of 39.3 Mb, and a longest scaffold of 47.5 Mb. Considering that the genome size is roughly 225 million bases with three chromosomes, the assembly statistics achieved for IndV4qm using IndV3 scaffolds can be considered very high-quality. The total genome size for IndV3 and IndV4qm after stitching chromosomes is 180 and 191 Mb, covering 80 and 90%, respectively of the estimated genome size. The linearity of the final chromosomes is validated using synteny to each other and to the other assemblies reported since the submission of this article. There are no major gaps in the synteny between IndV3s and chromosome-level genomes of *An. gambiae* and *An. funestus* (Supplementary Figures S7a/b, S7c/d), which show least shuffling of genes between the chromosomal arms of the three malaria vectors compared here.

The scaffold18 of IndV3 of length 3.8 Mb and the scaffold19 of PakV3 of length 4.2 Mb could not be uniquely assigned to chromosomal arms based on physical markers. A synteny between scaffold18 of IndV3 with the UCI genome suggests that these scaffolds span the centromere of chromosome 3 (Supplementary Figure S4) in the two assemblies. This also explains why markers from both 3L and 3R arms were present in these scaffolds. It should be mentioned that this region is represented by three scaffolds in IndV1 and two scaffolds in Waterhouse et al. (2020) assemblies with neither spanning the arms.

The quality of IndV3s depended on both the homology-based scaffolding method and the resolution of physical markers. The IndV4 assembly scaffolded using HiC data from the STE2 strain fixed many misoriented short scaffolds in IndV3s. In IndV4qm, within the 2Rb inversion locus, the assembly suggested a translocation (Figure 3C-middle) with the two inversion breakpoints mimicking inversion as shown by the orange arrow above the karyogram in Figure 3A. This perhaps is an optimized fit found by the SALSA tool to fit the conflicting HiC contacts from a heterozygous inversion. By simply shifting a block (10A2-13B3) in the direction of the blue arrow shown in both the karyogram in Figure 3A and in the contact map in Figure 3C-middle, the assembly was made linear to the physical markers as shown in Figure 3C-bottom. The correctness of this move is validated by the contact map shown in Figure 3C-bottom against STE-HiC data showing 2Rb inversion butterfly (circled).

A chromosome-level genome also provides a great resource to validate gene structures and study genome-wide gene expression across tissues and/or developmental stages. As shown in Supplementary Figure S8, there is a good correlation between various embryonic stages including PEM. Also, while there is good correlation between adult male and female, only adult females retain correlation with all early developmental stages. Not surprisingly, there is no correlation between post blood meal (PBM) ovary and caracasses left after reviving the PBM ovary (FeCa). We observe that the selected metabolic genes of interest, implicated in pyrethroid resistance in insects, *CYP6M10a* (David et al., 2014), *CYP6P4* (Bamou et al., 2019), and *CYP6P9a/b* (Weedall et al., 2019), are highly expressed in the larva stage compared to all other stages (Supplementary Figures S8a3–a5). This is expected because the majority of insecticides are targeted to control larvae. Also, *ACE1* and *KDR* genes, which are implicated in insecticide resistance, are differentially upregulated in larva with the exception of adult male where it is highly expressed (Supplementary Figures S8a1,a7). Interestingly, mutations in *KDR* and *ACE1* genes have been shown to impact male fitness (Weedall et al., 2019). The expression profile of the *SPO11* gene, with a role in meiosis, is detectably higher in the embryonic early stage (Supplementary Figure S8a12). *Cardinal* and *KH* genes, with the exception in adult female, display an inverse correlation across developmental stages. The expression profiles of *MRE11* and *RAD51* genes (Supplementary Figures S8a9,a11), known to form complexes, are highly correlated with high expression in PBM, perhaps suggesting a role in *Plasmodium* infection.

The 2Rb inversion polymorphism is reported to be more frequent in the type form of *An. stephensi*, in comparison to the intermediate and other forms. In the UCI strain, this inversion has become homozygous providing an opportunity to study the genetic makeup of this polymorphism. The 2Rb inversion reported by Mahmood and Sakai (1984) is from the Orangi collection and Karachi colony with 40% of individuals positive for this inversion (Mahmood and Sakai, 1984). Interestingly, the dissected female progenies from one family from the Karachi colony were all homozygous for the 2Rb inversion. We believe that the UCI strain, which is also homozygous for this inversion, may have originally been derived from this Karachi colony. We have analyzed and studied the genes within this inversion locus from the UCI assembly to obtain insight into the role of this polymorphism in adaptation to the environment, including insecticide resistance. The 2Rb inversion is associated with resistance to alphametrin (Mahmood and Sakai, 1984) and the circadian cycle (Ayala et al., 2014). There are over 1000 genes within this locus including one of the two major cytochrome P450 gene clusters. Among the cytochrome P450 genes in this locus, those associated with insecticide resistance are present in multiple copies, including CYP6a14 (four copies), CYP4c1 (five copies), and CYP4c21 (four copies) in Table 2. Many of these genes are reported to be implicated in resistance (David et al., 2013; Lien et al., 2019). Interestingly, according to David et al. (2013) alphametrin is approved for use by WHO in 1981–1984 in the same time frame when Mahmood and Sakai (1984) reported the homozygous inversion in individuals from the Karachi colony.

By comparing the genomes of the STE2, SDA-500, and UCI strains, we have shown that the diversity between them is 1 in 82 positions, which is very similar to the diversity of 1 in 68 seen and reported here between individuals from diverse geographical loci. Also, the diversity between the SDA-500 and UCI strains is 1 in 82, suggesting that the SDA-500 strain from Pakistan and the UCI strain are from different sources.

We believe that this is the first instance to obtain a useful chromosome level assembly from two draft assemblies of the same species using homology-based scaffolding followed by stitching using low-resolution physical marker data and/or HiC data from two strains. This is also the first chromosome-level assembly of a malaria vector from India. The assembly reported here offers a genomic context to genes and other genetic elements, such as 2Rb inversion locus, providing a framework for controlling malaria in India with state-of-the-art technologies, such as gene editing and gene drive.

## Materials and Methods

### Sources of Data Used in This Work

The draft assemblies of both the Indian (IndV1) and Pakistani (PakV1) strains of *An. stephensi* were downloaded from https://www.vectorbase.org/. The sequences are also available on GenBank under the accession ALPR00000000.

The transcriptome data was obtained from public sources (NCBI SRA database: SRP013839).

Individual insects for whole-genome sequencing was originally maintained at NIMR, Bangalore, and were obtained from Dr. Sushant K. Ghosh (see section “Acknowledgment”).

### Homology-Based Assembly

The *An. stephensi* genomes of the Indian strain (IndV1) and Pakistani strain SDA-500 (PakV1) were downloaded from https://www.vectorbase.org/.

Mate-pair libraries containing reads carry information about the arrangement/ordering of the chromosomes, which help to build scaffolds from the first-pass assembly that is obtained from deep sequencing of paired-end reads. This implies that the sequence information from the mate-pair reads are NOT directly used in the assembly, but only the knowledge of ordering information is inherited.

(a) Generation of read libraries
  Using the samtools wgsim (version 1.9), mate-pair libraries with varying insert sizes (1 kb, 5 kb, 10 kb, 50 kb, 100 kb, 500 kb, 1 Mb, 2 Mb, 5 Mb) and read length of 50 bp were simulated from PakV1 with flags set for zero base error rate, rate of mutation, and incorporation of indels.
(b) Confident set of reads for subsequent scaffolding
  To assess the quality of the reads generated from each library, they were mapped back against *An. stephensi* IndV1 genome using bowtie2 (version 2.3.5.1) (Langmead and Salzberg, 2012). Flags to suppress discordant and unpaired alignments were set. While processing the alignments, only paired reads mapped in the right orientation with the correct insert sizes were used for downstream analyses. Care was taken to remove multiple mapped reads from the concordant list of fastq reads.
(c) Scaffolding of pre-assembled contigs
  Reads from (b) were used for super-scaffolding of the IndV1 genome to get the IndV2 assembly using the SSPACE tool (version 3.0) (Boetzer et al., 2011). Now, from the IndV2 assembly, mate pairs with varying library sizes (1 kb, 5 kb, 10 kb, 50 kb, 100 kb, 500 kb, 1 Mb, 2 Mb, 5 Mb) were simulated again to get the reads concordantly mapping to PakV1 assembly. These reads were used to super-scaffold PakV1 to obtain the PakV2 assembly. This iterative procedure was continued till the L50 and N50 of the assemblies did not improve significantly with further iterations resulting in assemblies IndV3 for the Indian strain and PakV3 for the Pakistani strain (shown in Figure 1).

### Pseudomolecule Generation Using Physical Marker Data

DNA sequences for physical map data for all chromosomal arms were downloaded from the Supplementary Material of *An. stephensi* draft assembly paper (Jiang et al., 2014). These were subjected to analysis using BLAST under stringent conditions (blastn -query 3L.fa -db indV3 -outfmt 6 -evalue 1.0e-25 -max_target_seqs 1 -out 3L_indV3.out) against the assembled genome IndV3. The unique hit of the physical markers onto scaffold IDs was used as a measure of the quality of assembly at this stage. A Venn diagram was generated to check the assignment of physical markers to scaffolds of the assembly (Supplementary Figure S1a). The scaffold hitting against markers from two chromosomes is resolved using the number and linearity of markers, and in some cases using the level of homology at the DNA level. Chromosomes were put together after orienting the scaffolds using the order of the physical markers. 1000Ns were inserted to separate different scaffolds while stitching. Scaffolds in Supplementary Table S1 are those with doubtful orientation or overlapping markers.

The five chromosomal arms for the Indian strain are given below for a total genome size of 180 Mb, covering more than 80% of the estimated genome size.

• IndV3s 2L chromosome: f31 + f41 + r37 + r17 + f25 + r22 + f5 + f15 = 25,555,578 bases
• IndV3s 2R chromosome: f4 + f12 + r2 + r38 + f23 + f140 + f10 + r7 + f117 + r19 + r26 + f24 = 58,040,504 bases
• IndV3s 3L chromosome: f13 + f1 + f6 = 37,520,143 bases
• IndV3s 3R chromosome: r3 + r8 + f9 + f30 + f20 + f21 + f18 = 43,024,569 bases
• IndV3s X chromosome: r16 + r14 + f11 + f28 + f40 = 16,297,292 bases

The five chromosomal arms for the Pakistani strain are given below for a total genome size of 194 Mb, again covering more than 80% of the estimated genome size.

• PakV3s 2L chromosome: r44 + f50 + f45 + f21 + r33 + r6 + r14 = 22,755,475 bases
• PakV3s 2R chromosome: f4 + r12 + f1 + r5 + f43 + r27 + f24 + r9 + r20 + r29 = 62,559,946 bases
• PakV3s 3L chromosome: f19 + r15 + f10 + r3 + f25 + r7 = 40,347,823 bases
• PakV3s 3R chromosome: f2 + r28 + f8 + r38 + r16 + r34 + r22 + r23 + r72 + r84 + r19 + f71 = 51,425,578 bases
• PakV3s X chromosome: f17 + r13 + f11 + r41 + f51 = 16,419,921 bases

### Physical Map Conflict Resolution and Validation Using Synteny

The scaffolds that were mapped to markers from multiple chromosomes were assigned to unique chromosomes based on the number of mapped markers, percent identity, e-value, and alignment lengths. For example, Supplementary Table S1 shows scaffolds of IndV3s (ScaffoldID) assigned to markers from multiple chromosome arms (ChromosomeID) and that were resolved, as shown in column Assignment. All the validation by synteny of IndV3 reference genome assembly against PakV3s, *An. Gambiae*, and *An. funestus* genomes was done using SyMAP (version 4.2) (Soderlund et al., 2006, 2011).

### Generation of IndV4qm Reference

The IndV3 genome from the iterative approach was scaffolded by utilizing the HiC data from the UCI and STE2 strains, using SALSA (Ghurye et al., 2019a) to obtain two new assemblies called IndV4uci and IndV4ste2, respectively. As the orientation of the markers was similar in both the genomes, we further used QuickMerge (Chakraborty et al., 2016) to combine them and produced longer, continuous scaffolds, which resulted in the assembly IndV4qm. The scaffolds were then stitched into chromosomes based on the linearity of the physical markers. The contact map of the genome was produced using HiC-Pro (Servant et al., 2015). The Valid-Pairs output from HiC-Pro was converted into the required input format (.hic) of Juicebox (Durand et al., 2016) for visualization. The coordinates in the 2Rb inversion where translocation was observed were corrected by shifting and interchanging the location of the blocks between 17A3-13B5 and 13B3-10A2. By correcting this region, we were able to obtain an assembly where the physical markers in the 2R arm were in the expected linear order.

### Gene Annotation

AUGUSTUS (version 3.2.3) (Hoff and Stanke, 2019), a eukaryotic gene prediction tool, was used to find protein-coding genes in the IndV3 genome. AUGUSTUS uses *ab initio* gene prediction and reconciles the predicted gene structures with orthology to the proteome from a model organism. The model organism closest to *An. stephensi* was made available by AUGUSTUS for use in gene prediction is *Ae. aegypti.* AUGUSTUS provides a gff3 file delineating the exon–intron boundaries for each predicted gene along with its protein sequence. This gff file is uploaded to the browser, the link to which can be found in the section “Data Availability Statement.” The same gff3 file is used to compute gene expression profiles of all these genes across developmental stages.

### Identification of Genes of Interest

We used orthologs of the genes of interest from public databases by searching for the gene sequences from the closest available species. For the majority of genes, sequences were available in *An. gambiae*. However, for FREP1, no genes could be found in any related species. We took the amino acid sequence of the fibronectin domain of FREP1 from Niu et al. (2017) and found the full-length predicted gene containing this domain. The table presented in Supplementary Table S4 shows the database source, accession, and related organism used in the identification of these genes in *An. stephensi*.

### Finding Orthologs of ORs

To find the ORs from *An. stephensi* and *An. funestus*, fasta sequences of 79 OR genes reported for *An. gambiae* were used (Hill et al., 2002). Here, protein blast, blastp (version 2.7.1), was used for identifying orthologous genes from the predicted proteomes of *An. stephensi* and *An. funestus* using the 79 OR protein sequences from *An. gambiae* as query. The top hits are given in Supplementary Table S9. The amino acid sequences of ORs from *An. stephensi* and *An. funestus* were extracted from the respective proteomes, and tandem ORs were manually split and provided in Supplementary Text S2. Using clustalW (version 2.1), a multiple-sequence alignment of ORs from only *An. stephensi* and *An. funestus* was generated, because these two species are more closely related to each other than *An. gambiae* (Kamali et al., 2014). Using Figtree (version 1.4.4), we created phylogenetic trees shown in both Figure 6 and Supplementary Figure S5.

### Transcriptome Analysis

Developmental transcriptome data was downloaded from NCBI SRA (SRP013839). Mapping was done using transcriptome reads on IndV3s using the STAR aligner (version 2.6) (Supplementary Table S7). Read counts mapped per gene were obtained, and normalized RPKM was computed providing a normalized expression profile for all predicted genes across the developmental stages. The genes of interest and ORs were taken out from annotation files and used for plotting the line graph as well as in the heat map. The entire transcriptome analysis was done using R programming. Packages used in the analyses are pheatmap, stats, corrplot, knitr, ggplot2, dplyr, tidyr, and reshape2. A description of the functionality of each tool is given in Supplementary Table S8. For further details on the script and version of packages used, we provide a link to mark down the transcriptome report under the section “Data Availability Statement.”

### Isolation of Genomic DNA

Briefly, the insect tissue was homogenized, and the genomic DNA was isolated using Qiagen Genomic-tp (Qiagen). Later, fluorometric quantification of DNA was done using Qubit 2.0 (Invitrogen).

### Library Preparation and Sequencing

Whole-genome DNA libraries with an average insert size of 200 bp were made using NEBNext^®^ Ultra^TM^ II DNA Library Prep Kit for Illumina^®^ (New England Biolabs, 2016) using the protocol recommended by the company. Briefly, around 50 ng of DNA was used for library preparation, and DNA was sheared using Adaptive Focused Acoustic technology (Covaris, Inc.) to generate fragments of length around 200 bp. The fragments were end repaired, 3′-adenylated, ligated with Illumina adapters, and PCR enriched with Illumina sequencing indexes. The size selection was performed using solid-phase reversible immobilization (SPRI) beads (Agencourt AMPure XP Beads) from Beckman Coulter. The quality and quantity of the libraries were evaluated using Qubit (Invitrogen) and TapeStation (Agilent). The libraries were diluted and pooled with an equimolar concentration of each library. Cluster generation was done using cBot (Illumina) and paired-end sequenced on an Illumina HiSeq 2500 platform using TruSeq SBS Kit v3-HS (200 cycle) (Illumina, San Diego, CA) following the manufacturer’s recommendations.

### SNP Analysis for Genetic Diversity

DNA samples for seven individuals were extracted and sequenced for 30X coverage. Variant calling was performed using an in-house pipeline, which uses fastqc (version 0.11.5), bowtie2 (version 2.3.5.1), samtools (version 1.9), picard-tools (version 2.18.12), samtools (version 1.9) mpileup (Li et al., 2009), and bcftools (version 1.9) to extract variants. The resulting vcf files are filtered using bcftools (version 1.9) (Li, 2011) for variants above a quality of 10 and a minimum depth of three reads. Mapping percentages of the seven individuals on the IndV3 reference have been tabulated in Supplementary Table S6.

### JBrowse and Database

The entire web framework of the website was taken from MEGHAGEN LLC, which includes the pre-built platform with the capability of genome visualization, blast, and further bioinformatic analysis. The website is built using HTML, CSS, and javascript. The current website is hosted on Amazon Cloud running Linux EC2 instance provided by Meghagen LLC. It uses inbuilt servers (Apache, Shiny) to establish user side connections.

JBrowse is an open-source, fast, and full-featured genome browser built with JavaScript and HTML5. It is easily embedded into websites or apps but can also be served as a standalone web page (Buels et al., 2016). JBrowse can utilize multiple types of data in a variety of common genomic data formats, including genomic feature data in bioperl databases, GFF files, BED files, and quantitative data in wiggle files (Skinner and Holmes, 2010). The website for IndV3s here has been built using Apache/2.4.29 (Ubuntu), build 2020-03-13T12:26:16, and JBrowse version 1.16.8.

## Data Availability Statement

The improved IndV3s and PakV3s assemblies for India and SDA-500 are available on NCBI BioProject under the project ID PRJNA473393. Genome and gene annotation files reported here can be found at http://3.93.125.130/indv3s. Scripts and transcriptome related data can be found at https://github.com/SaurabhWhadgar/indv3stranscriptome.

## Author Contributions

AC: validation by synteny and resolving scaffold conflicts. SR: development of the homology-based assembly method and preparation of the manuscript. SJ: assembly and stitching of chromosomes. KP: annotation and genes of interest. JS: SNP analysis. CS: olfactory receptors. SW: transcriptome and browser/database (website). NK: DNA extraction from individuals and quantification. RR: sequencing. CG: maintenance of lab strains. BC: overseeing of lab work including library preparation and sequencing. SSu: initiation of the work, interpretation of results, and scientific editing of the manuscript. SSr: coming up with the concept, management of the team, and writing of the manuscript. All authors contributed to the article and approved the submitted version.

## Conflict of Interest

The authors declare that the research was conducted in the absence of any commercial or financial relationships that could be construed as a potential conflict of interest.

## References

[B1] Amichot M. Tarès S. Brun-Barale A. Arthaud L. Bride J. M. Bergé J. B. (2004). Point mutations associated with insecticide resistance in the *Drosophila* cytochrome P450 Cyp6a2 enable DDT metabolism. *Eur. J. Biochem.* 271 1250–1257. 10.1111/j.1432-1033.2004.04025.x 15030474

[B2] Ayala D. Ullastres A. González J. (2014). Adaptation through chromosomal inversions in Anopheles. *Front. Genet.* 5:129. 10.3389/fgene.2014.00129 24904633 PMC4033225

[B3] Bamou R. Sonhafouo-Chiana N. Mavridis K. Tchuinkam T. Wondji C. S. Vontas J. (2019). Status of insecticide resistance and its mechanisms in anopheles gambiae and anopheles coluzzii populations from forest settings in south cameroon. *Genes* 10:741. 10.3390/genes10100741 31554225 PMC6827028

[B4] Batzoglou S. (2005). The many faces of sequence alignment. *Brief. Bioinformatics* 6 6–22. 10.1093/bib/6.1.6 15826353

[B5] Boetzer M. Henkel C. V. Jansen H. J. Butler D. Pirovano W. (2011). Scaffolding pre-assembled contigs using SSPACE. *Bioinformatics* 27 578–579. 10.1093/bioinformatics/btq683 21149342

[B6] Bohbot J. Pitts R. J. Kwon H. W. Rützler M. Robertson H. M. Zwiebel L. J. (2007). Molecular characterization of the *Aedes aegypti* odorant receptor gene family. *Insect Mol. Biol.* 16 525–537. 10.1111/j.1365-2583.2007.00748.x 17635615 PMC3100214

[B7] Buels R. Yao E. Diesh C. M. Hayes R. D. Munoz-Torres M. Helt G. (2016). JBrowse: a dynamic web platform for genome visualization and analysis. *Genome Biol.* 17:66. 10.1186/s13059-016-0924-1 27072794 PMC4830012

[B8] Carlson J. R. (2001). Functional expression of a *Drosophila* odor receptor. *Proc. Natl. Acad. Sci. U.S.A.* 98 8936–8937. 10.1073/pnas.171311198 11481464 PMC55351

[B9] Chakraborty M. Baldwin-Brown J. G. Long A. D. Emerson J. J. (2016). Contiguous and accurate de novo assembly of metazoan genomes with modest long read coverage. *Nucleic Acids Res.* 44:e147. 10.1093/nar/gkw654 27458204 PMC5100563

[B10] Chakraborty M. Ramaiah A. Adolfi A. Halas P. Kaduskar B. Ngo L. T. (2020). Hidden features of the malaria vector mosquito, Anopheles stephensi, revealed by a high-quality reference genome. *bioRxiv* [Preprint]. 10.1101/2020.05.24.113019

[B11] Chida A. R. Ravi S. Jayaprasad S. Paul K. Saha J. Suresh C. (2020). A near-chromosome level genome assembly of Anopheles stephensi. *bioRxiv* [Preprint]. 10.1101/2020.04.27.063040 PMC7703621 33312190

[B12] David J.-P. Faucon F. Chandor-Proust A. Poupardin R. Riaz M. A. Bonin A. (2014). Comparative analysis of response to selection with three insecticides in the dengue mosquito *Aedes aegypti* using mRNA sequencing. *BMC Genomics* 15:174. 10.1186/1471-2164-15-174 24593293 PMC4029067

[B13] David J.-P. Ismail H. M. Chandor-Proust A. Paine M. J. I. (2013). Role of cytochrome P450s in insecticide resistance: impact on the control of mosquito-borne diseases and use of insecticides on Earth. *Philos. Trans. R. Soc. Lond., B, Biol. Sci.* 368:20120429. 10.1098/rstb.2012.0429 23297352 PMC3538419

[B14] Durand N. C. Robinson J. T. Shamim S. Machol I. Mesirov J. P. Lander E. S. (2016). Juicebox provides a visualization system for Hi-C contact maps with unlimited zoom. *Cell Syst.* 3 99–101. 10.1016/j.cels.2015.07.012 27467250 PMC5596920

[B15] Ghurye J. Koren S. Small S. T. Redmond S. Howell P. Phillippy A. M. (2019a). A chromosome-scale assembly of the major African malaria vector *Anopheles funestus*. *Gigascience* 8:giz063. 10.1093/gigascience/giz063 31157884 PMC6545970

[B16] Ghurye J. Rhie A. Walenz B. P. Schmitt A. Selvaraj S. Pop M. (2019b). Integrating Hi-C links with assembly graphs for chromosome-scale assembly. *PLoS Comput. Biol.* 15:e1007273. 10.1371/journal.pcbi.1007273 31433799 PMC6719893

[B17] Gnerre S. Lander E. S. Lindblad-Toh K. Jaffe D. B. (2009). Assisted assembly: how to improve a de novo genome assembly by using related species. *Genome Biol.* 10:R88. 10.1186/gb-2009-10-8-r88 19712469 PMC2745769

[B18] Hill C. A. Fox A. N. Pitts R. J. Kent L. B. Tan P. L. Chrystal M. A. (2002). G protein-coupled receptors in *Anopheles gambiae*. *Science* 298 176–178. 10.1126/science.1076196 12364795

[B19] Hoff K. J. Stanke M. (2019). Predicting genes in single genomes with Augustus. *Curr. Protoc. Bioinformatics* 65:e57. 10.1002/cpbi.57 30466165

[B20] Holt R. A. Subramanian G. M. Halpern A. Sutton G. G. Charlab R. Nusskern D. R. (2002). The genome sequence of the malaria mosquito *Anopheles gambiae*. *Science* 298 129–149. 10.1126/science.1076181 12364791

[B21] Jiang X. Peery A. Hall A. B. Sharma A. Chen X. G. Waterhouse R. M. (2014). Genome analysis of a major urban malaria vector mosquito, *Anopheles stephensi*. *Genome Biol.* 15: 459. 10.1186/s13059-014-0459-2 25244985 PMC4195908

[B22] Kamali M. Marek P. E. Peery A. Antonio-Nkondjio C. Ndo C. Tu Z. (2014). Multigene phylogenetics reveals temporal diversification of major African malaria vectors. *PLoS One* 9:e93580. 10.1371/journal.pone.0093580 24705448 PMC3976319

[B23] Kim J. Larkin D. M. Cai Q. Asan, Zhang Y. Ge R. L. (2013). Reference-assisted chromosome assembly. *Proc. Natl. Acad. Sci. U.S.A.* 110 1785–1790. 10.1073/pnas.1220349110 23307812 PMC3562798

[B24] Langmead B. Salzberg S. L. (2012). Fast gapped-read alignment with Bowtie 2. *Nat. Methods* 9 357–359. 10.1038/nmeth.1923 22388286 PMC3322381

[B25] Li H. (2011). A statistical framework for SNP calling, mutation discovery, association mapping and population genetical parameter estimation from sequencing data. *Bioinformatics* 27 2987–2993. 10.1093/bioinformatics/btr509 21903627 PMC3198575

[B26] Li H. (2018). Minimap2: pairwise alignment for nucleotide sequences. *Bioinformatics* 34 3094–3100. 10.1093/bioinformatics/bty191 29750242 PMC6137996

[B27] Li H. Handsaker B. Wysoker A. Fennell T. Ruan J. Homer N. (2009). The Sequence Alignment/Map format and SAMtools. *Bioinformatics* 25 2078–2079. 10.1093/bioinformatics/btp352 19505943 PMC2723002

[B28] Lien N. T. K. Ngoc N. T. H. Lan N. N. Hien N. T. Tung N. V. Ngan N. T. T. (2019). Transcriptome sequencing and analysis of changes associated with insecticide resistance in the dengue mosquito (*Aedes aegypti*) in Vietnam. *Am. J. Trop. Med. Hyg.* 100 1240–1248. 10.4269/ajtmh.18-0607 30834881 PMC6493926

[B29] Lukyanchikova V. Nuriddinov M. Belokopytova P. Liang J. Reijnders M. J. M. L. Ruzzante L. (2020). Anopheles mosquitoes revealed new principles of 3D genome organization in insects. *bioRxiv* [Preprint]. 10.1101/2020.05.26.114017 PMC9005712 35413948

[B30] Mahmood F. Sakai R. K. (1984). Inversion polymorphisms in natural populations of Anopheles stephensi. *Can. J. Genet. Cytol.* 26 538–546. 10.1139/g84-086 6498600

[B31] Neafsey D. E. Waterhouse R. M. Abai M. R. Aganezov S. S. Alekseyev M. A. Allen J. E. (2015). Mosquito genomics. Highly evolvable malaria vectors: the genomes of 16 Anopheles mosquitoes. *Science* 347:1258522. 10.1126/science.1258522 PMC4380271 25554792

[B32] Niu G. Franc̨a C. Zhang G. Roobsoong W. Nguitragool W. Wang X. (2017). The fibrinogen-like domain of FREP1 protein is a broad-spectrum malaria transmission-blocking vaccine antigen. *J. Biol. Chem.* 292, 11960–11969. 10.1074/jbc.M116.773564 28533429 PMC5512087

[B33] Schneeberger K. Ossowski S. Ott F. Klein J. D. Wang X. Lanz C. (2011). Reference-guided assembly of four diverse *Arabidopsis thaliana* genomes. *Proc. Natl. Acad. Sci. U.S.A.* 108 10249–10254. 10.1073/pnas.1107739108 21646520 PMC3121819

[B34] Servant N. Varoquaux N. Lajoie B. R. Viara E. Chen C. J. Vert J. P. (2015). HiC-Pro: an optimized and flexible pipeline for Hi-C data processing. *Genome Biol.* 16:259. 10.1186/s13059-015-0831-x 26619908 PMC4665391

[B35] Sharakhova M. V. Hammond M. P. Lobo N. F. Krzywinski J. Unger M. F. Hillenmeyer M. E. (2007). Update of the *Anopheles gambiae* PEST genome assembly. *Genome Biol.* 8:R5. 10.1186/gb-2007-8-1-r5 17210077 PMC1839121

[B36] Sharakhova M. V. Xia A. Tu Z. Shouche Y. Unger M. F. Sharakhov I. V. (2010). A physical map for an Asian malaria mosquito, *Anopheles stephensi*. *Am. J. Trop. Med. Hyg.* 83 1023–1027. 10.4269/ajtmh.2010.10-0366 21036831 PMC2963963

[B37] Skinner M. E. Holmes I. H. (2010). Setting up the JBrowse genome browser. *Curr. Protoc. Bioinformatics* Chapter 9:Unit9.13. 10.1002/0471250953.bi0913s32 21154710 PMC4350995

[B38] Soderlund C. Bomhoff M. Nelson W. M. (2011). SyMAP v3.4: a turnkey synteny system with application to plant genomes. *Nucleic Acids Res.* 39:e68. 10.1093/nar/gkr123 21398631 PMC3105427

[B39] Soderlund C. Nelson W. Shoemaker A. Paterson A. (2006). SyMAP: a system for discovering and viewing syntenic regions of FPC maps. *Genome Res.* 16 1159–1168. 10.1101/gr.5396706 16951135 PMC1557773

[B40] Vij S. Kuhl H. Kuznetsova I. S. Komissarov A. Yurchenko A. A. Van Heusden P. (2016). Chromosomal-level assembly of the Asian seabass genome using long sequence reads and multi-layered scaffolding. *PLoS Genet.* 12:e1005954. 10.1371/journal.pgen.1005954 27082250 PMC4833346

[B41] Waterhouse R. M. Aganezov S. Anselmetti Y. Lee J. Ruzzante L. Reijnders M. J. M. F. (2020). Evolutionary superscaffolding and chromosome anchoring to improve Anopheles genome assemblies. *BMC Biol.* 18:1. 10.1186/s12915-019-0728-3 31898513 PMC6939337

[B42] Weedall G. D. Mugenzi L. M. Menze B. D. Tchouakui M. Ibrahim S. S. Adjia N. A. (2019). A cytochrome P450 allele confers pyrethroid resistance on a major African malaria vector, reducing insecticide-treated bednet efficacy. *Sci. Transl. Med.* 11:eaat7386. 10.1126/scitranslmed.aat7386 30894503

[B43] Zimin A. V. Delcher A. L. Florea L. Kelley D. R. Schatz M. C. Puiu D. (2009). A whole-genome assembly of the domestic cow. *Bos taurus*. *Genome Biol.* 10:R42. 10.1186/gb-2009-10-4-r42 19393038 PMC2688933

